# Inflammation (IL-1β) Modifies the Effect of Vitamin D and Omega-3 Long Chain Polyunsaturated Fatty Acids on Core Symptoms of Autism Spectrum Disorder—An Exploratory Pilot Study ^†^

**DOI:** 10.3390/nu12030661

**Published:** 2020-02-28

**Authors:** Hajar Mazahery, Cathryn A. Conlon, Kathryn L. Beck, Owen Mugridge, Marlena C. Kruger, Welma Stonehouse, Carlos A. Camargo, Barbara J. Meyer, Bobby Tsang, Pamela R. von Hurst

**Affiliations:** 1 School of Sport, Exercise and Nutrition, College of Health, Massey University, Auckland 0745, New Zealand; h.mazahery@massey.ac.nz (H.M.); c.conlon@massey.ac.nz (C.A.C.); k.l.beck@massey.ac.nz (K.L.B.); o.mugridge@massey.ac.nz (O.M.); 2 School of Health Sciences, College of Health, Massey University, Palmerston North 4442, New Zealand; m.c.kruger@massey.ac.nz; 3 Riddet Institute, Massey University, Palmerston North 4442, New Zealand; 4 Commonwealth Scientific and Industrial Research Organisation (CSIRO), Adelaide, SA 5000, Australia; welma.stonehouse@csiro.au; 5 Department of Emergency Medicine, Massachusetts General Hospital, Harvard Medical School, Boston, MA 02114, USA; ccamargo@partners.org; 6 School of Medicine, Lipid Research Centre, Molecular Horizons, University of Wollongong, Illawarra Health and Medical Research Institute, Wollongong, NSW 2522, Australia; bmeyer@uow.edu.au; 7 Department of Paediatrics, Child and Youth Health, University of Auckland, Auckland 1023, New Zealand; bobby.tsang@waitematadhb.govt.nz

**Keywords:** autism, inflammation, interleukin 1, vitamin D, omega-3, intervention

## Abstract

Background: The role of vitamin D and omega-3 long chain polyunsaturated fatty acids (omega-3 LCPUFA) in improving core symptoms of autism spectrum disorder (ASD) in children has been investigated by a few randomised controlled trials and the results are mixed and inconclusive. The response to treatment with these nutrients is heterogenous and may be influenced by inflammatory state. As an exploratory analysis, we investigated whether inflammatory state would modulate the effect of these nutrients on core symptoms of ASD. Methods: Seventy-three New Zealand children with ASD (2.5–8.0 years) completed a 12-month randomised, double-blind, placebo-controlled trial of vitamin D (VID, 2000 IU/day), omega-3 LCPUFA; (OM, 722 mg/day docosahexaenoic acid), or both (VIDOM). Non-fasting baseline plasma interleukin-1β (IL-1β) was available for 67 children (VID = 15, OM = 21, VIDOM = 15, placebo = 16). Children were categorised as having undetectable/normal IL-1β (<3.2 pg/ml, *n* = 15) or elevated IL-1β (≥3.2 pg/mL, *n* = 52). The Social Responsiveness Scale (SRS) questionnaire was used to assess core symptoms of ASD (baseline, 12-month). Mixed model repeated measure analyses (including all children or only children with elevated IL-1β) were used. Results: We found evidence for an interaction between baseline IL-1β and treatment response for SRS-total, SRS-social communicative functioning, SRS-awareness and SRS-communication (all *P_interaction_* < 0.10). When all children were included in the analysis, two outcome comparisons (treatments vs. placebo) showed greater improvements: VID, no effect (all *P* > 0.10); OM and VIDOM (*P* = 0.01) for SRS-awareness. When only children with elevated IL-1β were included, five outcomes showed greater improvements: OM (*P* = 0.01) for SRS-total; OM (*P* = 0.03) for SRS-social communicative functioning; VID (*P* = 0.01), OM (*P* = 0.003) and VIDOM (*P* = 0.01) for SRS-awareness. Conclusion: Inflammatory state may have modulated responses to vitamin D and omega-3 LCPUFA intervention in children with ASD, suggesting children with elevated inflammation may benefit more from daily vitamin D and omega-3 LCPUFA supplementation.

## 1. Introduction

Autism spectrum disorder (ASD) is a complex neurodevelopment disorder with heterogeneous clinical presentation and aetiology. The diagnosis of ASD is based on the presence of impaired social and communicative functioning and repetitive and/or restricted interest and behaviours [1]. A large proportion of children with ASD have been reported to have co-occurring behavioural, medical and biological conditions [2,3,4]. Furthermore, growing evidence suggest an ongoing immune dysfunction in the brain and the peripheral immune system of a subset of individuals with ASD [5,6]. Several peripheral markers of inflammation (interleukin-1 beta (IL-1β), IL-6 and tumour necrosis factor-alpha (TNF-α)) have been found to be elevated in children with ASD [7,8,9,10,11].

Interleukin-1β, a major pro-inflammatory cytokine released from activated microglia (the unique resident cells of the central nervous system), and its receptors are found throughout the nervous system during critical developmental periods [12]. Peripheral and central IL-1β administration reduces neurogenesis and induces impaired social interaction, anxiety and stress which are symptoms associated with ASD [13,14,15]. In contrast, administration of IL-1 receptor antagonist (IL-1ra) reduces IL-1β induced impaired social interaction in animals [15]. 

Interleukin-1β has been frequently reported to be elevated in the plasma of children and adults with ASD [10,16,17]. Evidence also suggests that peripheral blood cells of individuals with ASD produce higher IL-1β levels at baseline and following stimulation (with lipopolysaccharide, LPS) [18,19]. Furthermore, genetic mutations and polymorphism in IL-1β and its receptors have been shown to be associated with ASD and cognitive performance [20,21]. Although, normal levels of IL-1β have been reported in post-mortem brains from ASD individuals [22], increased systemic levels can have adverse neurological consequences relevant to ASD because it can disrupt and cross the blood-brain barrier [23,24]. Ashwood et al. (2011) and Masi et al. (2017) reported a positive correlation between IL-1β (as well as other inflammatory markers) and more impaired behavioural outcomes and regressive onset in children with ASD [10,16]. 

It is generally agreed that ASD is driven by an interaction between genetic and non-genetic factors (such as nutritional factors) [25]. There is some data to support a role for vitamin D and omega-3 long chain polyunsaturated fatty acids (LCPUFA), especially eicosapentaenoic acid (EPA) and docosahexaenoic acid (DHA), in the development and management of ASD, though the findings are mixed [26,27]. These nutrients have immunomodulatory, anti-inflammatory and anti-oxidant properties, and both have important roles in brain function and structure and in the neurotransmitter system [26,27,28]. Several mechanistic pathways have been proposed for a role of vitamin D and omega-3 LCPUFA in inflammation. Vitamin D metabolites can exert their role in inflammation through their effect on protein expression of toll-like receptors 2 and 4, expression of phosphorylated signal transducer and activator of transcription and reactive oxygen species [29]. On the other hand, vitamin D deficiency has been suggested to be a direct consequence of an inflammatory condition or state [30]. Omega-3 LPUFAs have been shown to modulate inflammatory cytokines and immune function through different pathways, including inhibition of omega-6 derived pro-inflammatory eicosanoids (e.g., PGE2 and LTB4), enhancing the formation of several anti-inflammatory eicosanoids (e.g., resolvins and protectins), suppressing the activity of nuclear transcription factor, and reducing the pro-inflammatory enzymes and cytokines [31]. 

Evidence from animal studies suggests that prolonged exposure to vitamin D and/or omega-3 LCPUFA deficiency can lead to microglial activation and to an inflammatory and immunity state [28,32], as observed in children with ASD [33]. Furthermore, the deficiency in vitamin D and/or omega-3 LCPUFA leads to ASD-like behavioural symptoms [34,35], and supplementation with these nutrients ameliorates or rescues those behaviours in animal models of maternal inflammation [36,37]. Several reports from human studies also suggest that gestational vitamin D and/or omega-3 LCPUFA deficiency is associated with the development of ASD [38,39,40,41] and other psychological and neurodevelopmental disorders [42]. Furthermore, children with ASD have been shown to have inadequate intakes of vitamin D and omega-3 LCPUFA [11,12,13,14,15], as is reflected in significantly lower vitamin D and/or omega-3 LCPUFA status than their healthy counterparts [5,16].

The efficacy of vitamin D and omega-3 LCPUFA in improving ASD-related symptoms has been tested in a few randomised controlled trials. However, the results from these trials are mixed and inconclusive [26,27]. Using the Social Responsiveness Scale (SRS) (total and domains) questionnaire, our research group previously investigated the effect of vitamin D, omega-3 LCPUFA or both on core symptoms of ASD (VIDOMA trial) and also found mixed results [43]. Out of 21 outcome comparisons, only four showed a positive effect of treatment on core symptoms; two significant and two trends for greater improvements [43]. All four positive findings were attributed to omega-3 LCPUFA with or without vitamin D; vitamin D alone had no apparent effect. Furthermore, 20%–22% of children were positive responders (at least a 30% improvement in SRS-total).

The large variability in treatment response is a common challenge in ASD clinical trials [44]. Many factors (e.g., baseline severity, age, treatment dose, concurrent treatment/therapy, adherence and outcome measures) probably contribute to the variance in treatment response. It is important to note that some of these factors may also contribute to high placebo response, which is a significant confounding factor in ASD clinical trials [45]. However, other potential modifying factors (covariates) not identified previously may also contribute to this variance.

Although the mechanistic pathways by which these nutrients may affect ASD is not clear, it has been hypothesised that the relationship between these nutrients and ASD can be partly mediated by inflammatory markers, as seen in other neuropsychiatric conditions where inflammation may contribute [46,47,48]. To the best of our knowledge, no studies have examined the potential modifying effect of participants’ pre-treatment inflammatory state on vitamin D and omega-3 LCPUFA treatment response in children with ASD.

In this setting, we hypothesised that inflammation could modify the responsiveness to vitamin D or omega-3 LCPUFA intervention in children with ASD, such that there would be a greater improvement in core symptoms with vitamin D and omega-3 LCPUFA in the presence of inflammation.

## 2. Materials and Methods

### 2.1. Study Design and Participants

This study was conducted as an exploratory analysis of the data from the Vitamin D and Omega-3 LCPUFA in Autism (VIDOMA) trial. A detailed description of the design of the VIDOMA trial and its main results have been published [43,49,50]. The protocol was registered with the Australian New Zealand Clinical Trial Registry, ACTRN12615000144516, and the ethical approval was granted by Health and Disability Ethics Committee, NZ, Reference NO. 14/NTA/113. All parents/caregivers provided written informed consent. 

New Zealand children aged 2.5 to 8.0 years with confirmed medical diagnosis of ASD (confirmed by both a developmental paediatrician and in accordance with the criteria listed at DSM-5 [1]) and onset of symptoms after 18 months of age were recruited. Children were excluded if they: (1) were diagnosed as having developmental delay since birth, (2) failed to take corrective action for nutritional deficiencies identified at recruitment stage, or (3) had serum 25(OH)D ≥ 75 + 10 nmol/L (≥ 85 nmol/L) if they entered the trial in winter and ≥ 105 + 10 nmol/L (≥ 115 nmol/L) if they entered the trial in summer. 

Children who met the initial inclusion criteria had a blood draw and were screened for nutritional deficiencies (including vitamin D, iron and vitamin B_12_ deficiencies). Prior to randomisation, those deficiencies were addressed (refer to [49] and Table 1 for the list of deficiencies and the strategies used to address those deficiencies). Children were then randomly assigned to one of four treatment groups: vitamin D_3_ (VID, 2000 IU/day), omega-3 LCPUFA (OM, 722 mg/day DHA), both (VIDOM), or placebo. The treatment materials were delivered in 750 mg gel capsules with a tear-off nozzle manufactured and supplied by Douglas Nutrition Ltd (Auckland, NZ). All study capsules were identical in appearance and were tasteless and colourless. A third party not involved in any aspects of the study was responsible for generating the randomisation sequence using Website Randomization.com (http://www.randomization.com/) and random block design in blocks of 4 and 8. Randomisation was stratified by age (2.5–5.0 and 5.0–8.0 years old age groups) and severity of ASD (based on confirmed medical diagnosis; mild, moderate, severe). Researchers, children, and caregivers were blinded to treatment allocations until after data analysis.

### 2.2. Measurements

The outcome measures were the severity and core symptoms of ASD assessed at baseline and 12 months, and a standardised psychological test (SRS-total and domains, described below) [52] was used to assess these outcomes. Standardised instructions were given to all caregivers on how to complete the questionnaire during their visit to the Human Nutrition Research Unit (HNRU). 

The Social Responsiveness Scale^TM^, Second Edition (SRS-2) [52] is a quantitative approach to screen and diagnose social impairment and repetitive behaviour as a single trait in ASD. SRS provides scores for five treatment subscales (social awareness, social cognition, social communication, social motivation, and repetitive and restricted interests and behaviours [RRB]) as well as scores for two DSM-5 compatible subscales, social communicative functioning (the composite score of social awareness, social cognition, social communication and social motivation) and RRB. The SRS has been found to discriminate children with ASD from typically developing children, from children with oppositional defiant/conduct disorder, and from other psychiatric patients with/without pervasive developmental disorders [53]. In a cross-cultural validity study of the SRS, Bolte et al. (2008) reported a good internal consistency, test–retest reliability and inter-rater reliability, and a good convergent validity with other ASD diagnostic tools [54].

Demographics, medical history, and anthropometric measures (weight and height) were collected at baseline. Caregivers also completed weekly online surveys to collect information regarding medication use, therapy and compliance. Compliance was calculated using cumulative pill counts at the end of the study, and adherence was measured as a percentage; (number of pills supplied minus number of pills not taken)/number of pills supplied × 100. Compliance was also confirmed by biomarker analysis (serum 25(OH)D and red blood cell omega-3 index). 

### 2.3. Biochemical Analysis

Nutritional biomarkers, including 25-hydroxyvitamin D, 25(OH)D; red blood cell (RBC) fatty acids; IL-1β; calcium; albumin; iron; and vitamin B_12_ were assayed from a non-fasted venous blood sample. The blood was protected from light and allowed to clot for 30 min and centrifuged for 10 min at 2000 rpm at 4 °C within 2 h of sampling. For detailed information regarding the assays and methods refer to the study protocol paper [49].

IL-1β was assayed using spare samples collected at baseline and stored at −80 °C. IL-1β was measured from thawed serum (at room temperature) by Abcam IL-1β ELISA kit according to the manufacturer’s instructions. In this assay, an antibody specific for IL-1β was coated onto the wells of the microtiter plates. Samples, including standards of known IL-1β concentrations and unknowns were pipetted into these wells and incubated at room temperature. The wells were then washed and a biotinylated antibody specific for IL-1β was added to the wells and incubated. After further washing, a streptavidin-peroxydase conjugate was added to each well and incubated. The wells were then washed to remove all unbound enzymes and TMB solution, which acts on the bound enzyme was added to induce a coloured reaction product. The intensity of this coloured product was directly proportional to the concentration of IL-1β present in the samples. 

### 2.4. Statistical Analysis

Sample size was calculated a priori for the primary objective of the original study (VIDOMA trial), change in core symptoms in children with ASD, therefore no sample size calculation was performed for the current exploratory analysis. 

Statistical analysis was performed using IBM SPSS version 25.0 (Armonk, NY, USA). A two-sided *P* < 0.05 was considered statistically significant, and a *P* < 0.10 (borderline significant) suggestive of a trend for treatment effect. No adjustment was made for multiple outcome measures comparisons. A partial eta-squared (*η_p_^2^)* of 0.01, 0.06, and 0.14 was considered small, medium and large effect size, respectively. The variables were tested for normality using the Kolmogorov–Smirnov, Shapiro–Wilk tests and normality plots. Non-normally distributed data were transformed into approximate normal distributions by logarithmic transformations. The data were reported as mean ± SD for normally distributed data, as median (25th, 75th percentiles) for non-normally distributed data, and as frequencies for categorical data. 

Participants were categorised as either having undetectable/normal IL-1β (within the first quartile <3.2 pg/mL) or elevated IL-1β (within the second/third/fourth quartiles ≥3.2 pg/mL). The cut-off of 3.2 pg/mL was found to be comparable to the mean levels reported in populations with inflammation [55]. Baseline socio-demographic and behavioural characteristics and biochemical markers (serum 25(OH)D and omega-3 index defined as RBC DHA + EPA) were compared across IL-1β categories using independent t-test and Wilcoxon signed-rank (continuous variables) and chi-square tests (categorical variables). 

We investigated the modifying effect of baseline IL-1β on behavioural response to intervention using the mixed effects model analysis adding the interaction term “baseline IL-1β x treatment groups”. We found evidence for an effect of interaction on treatment response for several behavioural outcomes (See Results section). Accordingly, two mixed effects models for each behavioural outcome were used to compare the effects of treatment on ASD behavioural outcomes (SRS-total and domains over time). Mixed effects model 1 included all children regardless of their inflammatory states at baseline, and mixed effects model 2 included only children with elevated IL-1β at baseline. Treatment (VID vs. placebo, OM vs. placebo, and VIDOM vs. placebo) and time (baseline and 12 months) were included as fixed effects. We found no effect of baseline ASD severity on treatment response. Covariates included in the models were compliance, medication use and therapy over the study period. Analysis was conducted on children who completed the trial and whose serum IL-1β was available at baseline. The results of the two mixed effects models were compared. 

## 3. Results

Seventy-three children out of 117 initial recruits completed the trial. The baseline characteristics of these children are reported elsewhere [43]. Of these 73 children, baseline serum IL-1β was available for 67 children, who comprised the analytical sample for the current analysis (vitamin D, 15; omega-3, 21; vitamin D + omega-3, 15; placebo, 16). The mean age of these children was 5.3 ± 1.4 years. Participants included 56 boys (84%), 34 New Zealand European (52%), and 23 overweight/obese (34%). There were no significant differences in baseline characteristics between children with and without inflammation (Table 2) or between treatment groups at baseline. 

**Figure 1:**
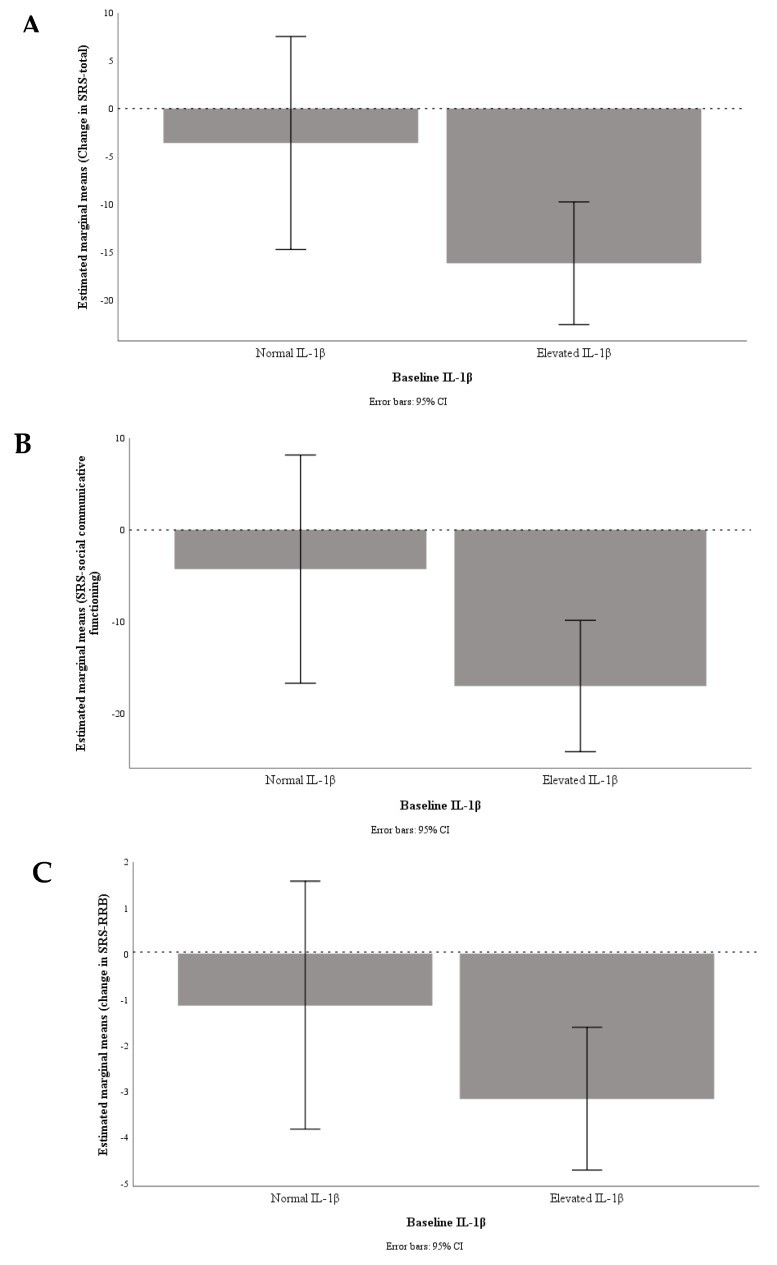
Graphical presentation of change in SRS-total (**A**), SRS-social communicative functioning (**B**), and SRS-repetitive and restricted interests and behaviours (RRB) (**C**) by baseline inflammation status; undetectable/normal, IL-1 β < 3.2 pg/mL (*n* = 14) vs. elevated, IL-1β ≥ 3.2 pg/mL (*n* = 37).

Out of 67 children, 52 (VID 9, OM 16, VIDOM 12, and placebo 15) had elevated IL-1β at baseline. We found a trend for greater improvement in SRS-total (−17 ± 21 vs. −4.5 ± 21, *P* = 0.08 and *η_p_^2^* = 0.07), SRS-social communicative functioning (−16 ± 19 vs. −3.4 ± 18, *P* = 0.056 and *η_p_^2^* = 0.08) and SRS-RRB (-3.1 ± 5.0 vs. −1.3 ± 3.7, *P* = 0.19 and *η_p_^2^* = 0.04) in children with elevated IL-1β at baseline (*n* = 37, active treatments minus placebo) than children with normal IL-1β (*n* = 14) (Figure 1). 

We found evidence for an effect of interaction (with baseline inflammatory state) on treatment response for SRS-total (*P* = 0.06), SRS-social communicative functioning (*P* = 0.04), SRS-awareness (*P* = 0.006), and SRS-communication (*P* = 0.09). Of 21 outcome measure comparisons (interventions vs. placebo), four (two significant and two borderline significant) showed greater improvements when all children (regardless of the presence/absence of inflammation at baseline) were included, and 10 (five significant and five borderline significant) showed greater improvements when only children with elevated IL-1β were included. In the mixed model analysis 1 when all children were included (Table 3), we found that VID was associated with no effect on behavioural outcomes (all *P* > 0.1 and negligible effect sizes), OM was associated with a greater improvement in SRS-awareness (*P* = 0.01 and *η_p_^2^* = 11) and a trend for greater improvement in SRS-total (*P* = 0.06 and *η_p_^2^* = 0.07), and VIDOM with a greater improvement in SRS-awareness (*P* = 0.01 and *η_p_^2^* = 0.11) and a trend for greater improvement in SRS-social communicative functioning (*P* = 0.05 and *η_p_^2^* = 0.07) compared with placebo (not adjusted for multiple outcome measure comparisons). However, in mixed model analysis 2 when only children with elevated IL-1β at baseline were included (Table 4), we found that VID was associated with a greater improvement in SRS-awareness (*P* = 0.01 and *η_p_^2^* = 0.14) and a trend for greater improvement in SRS-total (*P* = 0.07 and *η_p_^2^* = 0.08), SRS-communication (*P* = 0.07 and *η_p_^2^* = 0.07) and SRS-social communicative functioning (*P* = 0.09 and *η_p_^2^* = 0.07), OM was associated with a greater improvement in SRS-awareness (*P* = 0.003 and *η_p_^2^* = 0.18), SRS-total (*P* = 0.01 and *η_p_^2^* = 0.14), SRS-social communicative functioning (*P* = 0.03 and *η_p_^2^* = 0.11) and a trend for greater improvement in SRS-motivation (*P* = 0.05 and *η_p_^2^* = 0.09), and VIDOM with a greater improvement in SRS-awareness (*P* = 0.01 and *η_p_^2^* = 0.14) and a trend for greater improvement in SRS-social communicative functioning (*P* = 0.05 and *η_p_^2^* = 0.09) compared with placebo (not adjusted for multiple outcome measures comparisons). Due to small sample size, no subgroup analysis was performed for those with undetectable/normal IL-1β.

## 4. Discussion

We investigated whether pre-treatment blood IL-1β concentrations predicted vitamin D or omega-3 LCPUFA treatment response. Despite preclinical evidence indicating that vitamin D and omega-3 LCPUFA may be promising dietary supplements for managing both core and associated symptoms of ASD, the findings of prior vitamin D and omega-3 LCPUFA clinical trials have been mixed and inconclusive [26,27,43]. ASD is a heterogenous disorder, with a large variability in pro- and anti-inflammatory states [56]. Together with the well-documented immunomodulatory properties of vitamin D and omega-3 LCPUFA [29,31], we hypothesised that the inconsistent findings of recent vitamin D and omega-3 clinical trials might be attributed, at least in part, to the variability in participants’ pre-treatment inflammatory state. If vitamin D and omega-3 LCPUFA supplementation only benefits children with inflammation as part of their syndrome, then there can be: (1) only a small effect size improvement in symptoms of ASD with supplementation for a heterogeneous cohort of children with ASD, and (2) a large effect size improvement with supplementation for a homogenous subset of children, because it is effective for most of this subset.

In a 12-month, double-blind, randomised, placebo-controlled trial (VIDOMA trial), we previously found evidence for a greater improvement with omega-3 LCPUFA with or without vitamin D in social interaction in children with ASD compared with placebo but the difference did not reach statistical significance (*P* < 0.05) for most comparisons. In this exploratory analysis of data from the VIDOMA trial, we found that both vitamin D and omega-3, either individually or together, were significantly superior over placebo in improving several domains of social and communicative functioning when pre-treatment blood IL-1β concentrations were elevated. We confirmed that failure to include participants’ pre-treatment inflammatory state in our statistical model resulted in weak or null findings, whereas inclusion of participants’ pre-treatment inflammatory state improved the power of our model and resulted in more positive findings. 

To the best of our knowledge, there are no ASD clinical trials that have investigated the modifying effect of pre-treatment inflammatory state on vitamin D and omega-3 LCPUFA treatment response. However, our finding is consistent with that of one open label ASD clinical trial that investigated the role of other dietary supplements with immunomodulating properties (luteolin) and considered the effect of participants’ pre-treatment inflammatory state on their findings. Tsilioni et al. (2015) showed a greater improvement with luteolin in all domains of the Vineland Adaptive Behaviour Scale (VABS) in 4–10 years old children with ASD with a high baseline inflammatory state (elevated IL-6 and TNF in 10 children) as compared to a low inflammatory state [57]. 

Further evidence comes from an omega-3 LCPUFA clinical trial in depression (a disease with an established inflammatory component). Mazereeuw et al. (2017) [58] investigated the effect of omega-3 LCPUFA on depressive symptoms among 155 participants with major depressive disorder (MDD). The authors found negligible overall treatment group differences, however, when participants were stratified based on their inflammatory state at baseline (IL-1ra, IL-6, and hs-CRP), participants with any high inflammatory state improved more on active treatment than placebo. The active-placebo difference increased with increasing numbers of markers of high inflammation, a finding suggestive of identification of a more homogenous cohort of participants when multiple markers of inflammation are employed. It is important to note that IL-1ra, one of the inflammatory markers measured in this study, is a natural inhibitor of the pro-inflammatory effect of IL-1β, and has been shown to be elevated in subjects with ASD [59]. Elevated IL-1ra may be part of a homeostatic attempt to counteract an inflammatory state.

Finally, further evidence for a role of baseline inflammatory state on treatment response come from trials that investigated the treatment response to a range of types of antidepressants in populations with depression [60], to anti-psychotic treatments in populations with psychosis [61], and to behavioural therapies in populations with chronic pain [62]. These trials were consistent in showing that where treatments have immune-modulating properties, participants with the higher inflammatory state are treatment responders. By contrast, where the treatment has no immune-modulating effect, participants with a higher inflammatory state are non-responders. 

Mazereeuw et al. (2017) [58] reported that participants with a high pre-treatment inflammatory state were less placebo responsive than participants with a low inflammatory state (with a medium to large effect size difference), a finding confirmed by others [63]. The mechanism by which systemic inflammation reduces placebo response is not well understood. However, it has been suggested that social withdrawal, which is a core symptom of ASD, is also a core behaviour induced by inflammatory state [64]. These findings raise the possibility that chronic inflammatory activity may impair an individual’s ability to access social perceptions and emotions that are essential for placebo response. Unfortunately, we were unable to assess the effect of baseline inflammatory state on placebo response in our exploratory analysis because almost all children in the placebo group (with the exception of one child) were identified as having elevated IL-1β, hence such comparisons were not possible.

Immune system alterations have been demonstrated in both cerebrospinal fluid and peripheral blood of populations with ASD [10,16,17]. Children with ASD, at least a subset of individuals, have been demonstrated to have elevated pro-inflammatory cytokines such as IL-1α and β, IL-1Ra, IL-4, IL-6, IL-10, TNF-α, and IFN-γ [10,16,17,65]. Furthermore, elevated pro-inflammatory cytokines such as IL-1β, IL-6, and TNF-α has been shown to positively correlate with more severe behavioural outcomes [10,16,65]. Evidence from experimental studies show that experimentally-induced acute inflammation impairs cognitive functioning and emotional processing [66,67]. Therefore, immune imbalance arises as a possible pathway for anti-inflammatory drug intervention (reviewed by Marchezan et al. (2018) [68]). Several therapies with primary anti-inflammatory and immunomodulatory actions (e.g., Celecoxib) and therapies that display a capacity for immunomodulatory effect (e.g., vitamin D and omega-3 LCPUFA) have been investigated in populations with ASD [43,69]. Undesired pro-inflammatory gastrointestinal side effects of anti-inflammatory treatments [70] have been discussed as a limitation of such therapies, which may also result in heterogeneity in treatment response. In light of the high prevalence of gastrointestinal disorders in children with ASD [71], finding therapies with little or no gastrointestinal adverse effects is of high importance. Vitamin D and omega-3 LCPUFA have been shown to positively affect gut microbial communities, mucosal homeostasis, and gut inflammation as well as systemic inflammation [29,31,72,73]. Accordingly, these nutrients might be considered good candidates to address inflammation in children with ASD.

The results from the present study indicate that vitamin D and omega-3 LCPUFA have the potential to enhance social and communicative functioning in children with ASD, particularly when a priori stratification of children with ASD into treatment conditions are based on known pre-treatment inflammatory state. However, the findings must be interpreted with several potential limitations kept in mind.

This study is limited to reporting the findings of an exploratory analysis of the VIDOMA trial. In the absence of multiple measurements of cytokines, it does not directly address the molecular mechanisms underlying the observed effects. Also, because the collection of endpoint spare blood samples was not a priority, they were available for a very limited number of children. Therefore, we were unable to assess the effect of intervention on inflammatory state after 12 months and to investigate the relationship between the change in inflammatory state and the change in behavioural outcomes. Participants with and without spare blood samples were comparable on ASD severity, treatment allocation, and treatment outcome, suggesting no selection bias. Replication is a necessary step before a firm conclusion is made. Future studies should test the replicability of these exploratory findings, considering multiple markers of inflammation. Employing multiple markers may facilitate identification of a more homogeneous cohort of subjects with ASD positively responding to vitamin D and omega-3 LCPUFA versus placebo. 

Our choice of IL-1β as a marker of inflammation was based on reviews of literature reporting inflammatory markers in ASD [10,16,17], the impact of vitamin D and omega-3 LCPUFA on inflammatory markers [28,32,74], and the stability of the inflammatory markers in the blood [75]. It has been suggested that IL-1β is degraded within 2–3 years of storage. The time-lag between baseline blood collection (2015–2016) and blood analysis for inflammatory marker (end of 2018) was 2–3 years, and the possibility of IL-1β degradation cannot be ruled out (an underestimation of levels in our children with ASD). Also, because the measurement of inflammatory markers was not the primary objective of the VIDOMA trial, the preparation of participants (fasting state, blood collection timing, and consideration of prior physical activity) for the measurement of inflammatory markers might have not been in line with recommendations/guidelines. However, samples were stored at −80 °C and were not thawed and re-frozen. Future studies with the aim of stratification based on inflammatory markers should consider the strict guidelines to have a more reliable measure of inflammatory markers [75].

Finally, there is no criteria/cut-off for inflammation using IL-1β. However, in our study we used a cut-off based on the first quartiles vs. other quartiles for stratification. Our cut-off value of 3.2 pg/mL is consistent with the levels reported in populations with inflammation [55]. 

## 5. Conclusions

The present study provides supporting evidence for the possible beneficial role of vitamin D and omega-3 LCPUFA in managing some core symptoms of ASD in the subgroup of children with inflammation. Together with the results of several clinical trials showing a modifying effect of participants’ pre-treatment inflammatory state on treatment response, and clinical trials reporting the safety of vitamin D and omega-3 LCPUFA treatment in children with ASD [43], these findings justify further large-scale studies to validate the effects of vitamin D and omega-3 LCPUFA in children with ASD stratified based on their pre-treatment inflammatory state. 

## Figures and Tables

**Table 1:** Nutritional deficiencies and their management strategies prior to entering the intervention trial.

Nutritional Deficiency	Management
Vitamin D	Participants with serum 25(OH)D concentrations <25 nmol/L were offered supplementation of 400IU per day ^1^
Iron	Children with iron deficiency were offered iron supplements and postponed entry into the trial after 3 months. Children were retested. A child was iron deficient when 2 of the following pools were abnormal: red cell pool (haemoglobin <111 gr/L, red blood cell distribution width >14%), transport iron (iron saturation <16%) and/or storage iron (serum ferritin ≤15 µg/L) ^1^. Criteria for treatment were according to the New Zealand Ministry of Health guidelines ^2^
Vitamin B_12_	Children with serum levels <110 pmol/L were offered the option of prescribed supplements or dietary advice to improve status

^1^ New Zealand Ministry of Health 2015 [51]. ^2^ Retrieved from https://www.starship.org.nz/for-health-professionals/starship-clinical-guidelines/i/iron-deficiency/ on 5th of March 2015.

**Table 2:** The relationship of interleukin 1 beta (IL-1β) with biochemical markers and behaviours in 67 children with autism spectrum disorder (ASD).

Variables	Total (*n* = 67)	IL-1β		
		Undetectable/Normal (*n* = 15)	Elevated (*n* = 52)	*p*-Value *
Age (years), mean ± SD	5.3 ± 1.4	5.5 ± 1.4	5.3 ± 1.4	0.61
Sex, *n* (%)				0.67
Male	56 (84)	12 (21)	44 (79)	
Female	11 (16)	3 (27)	8 (73)	
Ethnicity, *n* (%)				0.64
New Zealand European	34 (52)	10 (29)	24 (71)	
Māori	9 (14)	2 (22)	7 (78)	
Pacific	2 (3)	0 (0)	2 (100)	
Asian	10 (15)	1 (10)	9 (90)	
Others	11 (17)	2 (18)	9 (82)	
Annual household income (NZ$), *n* (%)				0.24
<60,000	15 (25)	1 (7)	14 (93)	
60,000–120,000	32 (53)	8 (25)	24 (75)	
>120,000	13 (22)	4 (31)	9 (69)	
Season of blood collection, *n* (%)				0.25
Summer and Autumn	36 (54)	10 (28)	26 (72)	
Winter and Spring	31 (46)	5 (16)	26 (84)	
BMI-for-age categories, *n* (%)				0.93
Normal (<85th percentile)	44 (66)	10 (23)	34 (77)	
Overweight/obese (≥85th percentiles)	23 (34)	5 (22)	18 (78)	
Severity of ASD (based on confirmed medical diagnosis), *n* (%)				0.29
Mild	29 (43)	9 (31)	20 (69)	
Moderate	29 (43)	4 (14)	25 (86)	
Severe	9 (14)	2 (22)	7 (78)	
Scores on ASD behavioural symptoms (assessed using SRS), mean ± SD				
Total	101 ± 26	98 ± 26	102 ± 27	0.66
Social and communicative functioning	82 ± 22	79 ± 22	83 ± 22	0.55
Awareness	13 ± 4.0	14 ± 4.0	13 ± 4.0	0.64
Cognition	20 ± 5.7	19 ± 5.9	21 ± 5.6	0.26
Communication	34 ± 10	33 ± 10	34 ± 10	0.89
Motivation	14 ± 5.6	13 ± 4.2	15 ± 5.9	0.14
RRB	20 ± 6.0	20 ± 5.1	20 ± 6.3	0.93
Biochemical markers				
Serum 25(OH)D (nmol/L), mean ± SD	62 ± 24	66 ± 21	61 ± 25	0.41
Omega-3 index **, median (25th, 75th percentiles)	4.7 (4.4, 5.2)	4.7 (4.3, 6.2)	4.7 (4.4, 5.3)	0.55

ASD, autism spectrum disorder; IL-1β, interleukin 1 beta; SRS, Social Responsiveness Scale; RRB, repetitive and restricted interests and behavior. * Independent sample t-test for normally distributed data and chi-square for categorical variables were performed, unless otherwise stated. ** Mann Whitney U Test.

**Table 3:** Core symptoms of ASD assessed using the Social Responsiveness Scale (SRS) among children whose IL-1β was available at baseline and who completed the study across treatment groups (*n* = 67).

Outcome Variables	Study Groups			
	VID (*n* = 15)	OM (*n* = 21)	VIDOM (*n* = 15)	Placebo (*n* = 16)
Total				
Baseline	100 ± 24	100 ± 26	97 ± 29	108 ± 27
Endpoint	92 ± 32	82 ± 31	84 ± 33	102 ± 24
Change	−8.6 ± 25	−18±18	−13 ± 21	−5.8 ± 12
*P*-value ^1^	0.56	0.06	0.11	
Effect size	<0.01	0.07	0.04	
Social communicative functioning				
Baseline	82 ± 20	81 ± 21	78 ± 23	88 ± 24
Endpoint	74 ± 26	67 ± 25	62 ± 28	82 ± 19
Change	−7.8 ± 20	−14 ± 16	−16 ± 24	−5.6 ± 11
*P*-value ^1^	0.62	0.11	0.05	
Effect size	<0.01	0.05	0.07	
Social awareness				
Baseline	13 ± 2.9	13 ± 3.9	13 ± 4.4	13 ± 4.8
Endpoint	13 ± 3.5	12 ± 4.3	11 ± 5.1	13 ± 4.0
Change	−0.5 ± 2.7	−1.6 ± 2.4	−1.7 ± 3.5	0.4 ± 2.8
*P*-value ^1^	0.26	0.01	0.01	
Effect size	0.02	0.11	0.11	
Social cognition				
Baseline	20 ± 5.8	20 ± 5.4	19 ± 6.6	22 ± 5.1
Endpoint	18 ± 7.9	19 ± 14	17 ± 7.0	20 ± 5.5
Change	−1.4 ± 4.8	−0.9 ± 12	−2.3 ± 4.1	−2.3 ± 2.8
*P*-value ^1^	>0.1	>0.1	>0.1	
Effect size ^2^	<0.01	<0.01	<0.01	
Communication				
Baseline	34 ± 9.8	32 ± 9.5	33 ± 12	37 ± 11
Endpoint	30 ± 12	26 ± 11	28 ± 12	34 ± 9.2
Change	−3.9 ± 10	−5.8 ± 8.1	−5.3 ± 9.8	−2.4 ± 7.1
*P*-value ^1^	>0.1	>0.1	>0.1	
Effect size ^2^	0.01	0.04	0.05	
Social motivation				
Baseline	15 ± 5.4	15 ± 5.5	13 ± 5.3	16 ± 6.2
Endpoint	13 ± 5.9	11 ± 5.6	11 ± 5.5	14 ± 4.0
Change	−1.9 ± 4.7	−3.8 ± 4.3	−2.0 ± 4.5	−1.1 ± 4.3
*P*-value ^1^	>0.1	>0.1	>0.1	
Effect size ^2^	<0.01	0.04	0.01	
Repetitive and restricted interest and behaviour				
Baseline	20 ± 6.0	19 ± 6.6	18 ± 5.6	22 ± 5.7
Endpoint	18 ± 6.6	16 ± 7.8	17 ± 7.0	20 ± 5.5
Change	−2.7 ± 5.1	−3.5 ± 4.3	−1.4 ± 4.9	−1.8 ± 5.4
*P*-value ^1^	>0.1	>0.1	>0.1	
Effect size ^2^	<0.01	0.02	<0.01	

VID, vitamin D; OM, omega-3; VIDOM, vitamin D + omega-3; SRS, Social Responsiveness Scale. Values are reported as mean ± SD. ^1^ Pair-wise mixed effects models. The analyses were adjusted for therapy, medication, and compliance over the study period. ^2^ Partial eta-squared η_p_^2^; 0.01 = small effect size; 0.06 = medium effect size; 0.14 = large effect size.

**Table 4:** Core symptoms of ASD assessed using the Social Responsiveness Scale (SRS) among children whose IL-1β was elevated (IL-1β ≥ 3.2 pg/mL) and who completed the study across treatment groups (*n* = 52).

Outcome Variables	Study Groups			
	VID (*n* = 9)	OM (*n* = 16)	VIDOM (*n* = 12)	Placebo (*n* = 15)
Total				
Baseline	98 ± 24	104 ± 27	97 ± 29	106 ± 28
Endpoint	83 ± 32	82 ± 33	86 ± 33	100 ± 23
Change	−15 ± 23	−21 ± 19	−11 ± 23	−6.3 ± 13
*P*-value ^1^	0.07	0.01	0.17	
Effect size ^2^	0.08	0.14	0.04	
Social communicative functioning				
Baseline	81 ± 21	84 ± 21	78 ± 23	86 ± 24
Endpoint	67 ± 27	66 ± 26	63 ± 28	80 ± 19
Change	−14 ± 17	−17 ± 16	−15 ± 26	−6.1 ± 11
*P*-value ^1^	0.09	0.03	0.05	
Effect size ^2^	0.07	0.11	0.09	
Social awareness				
Baseline	13 ± 2.3	14 ± 4.1	13 ± 4.4	13 ± 5.0
Endpoint	12 ± 3.6	12 ± 4.4	12 ± 5.2	13 ± 4.0
Change	−1.4 ± 2.7	−1.8 ± 2.5	−1.0 ± 2.6	0.5 ± 2.9
*P*-value ^1^	0.01	0.003	0.01	
Effect size ^2^	0.14	0.18	0.14	
Social cognition				
Baseline	19 ± 4.9	21 ± 5.9	21 ± 6.7	22 ± 5.0
Endpoint	16 ± 7.9	19 ± 16	18 ± 7.3	19 ± 6.0
Change	−3.1 ± 4.3	−1.6 ± 13	−2.6 ± 4.4	−2.2 ± 2.9
*P*-value ^1^	>0.1	>0.1	>0.1	
Effect size ^2^	<0.01	<0.01	<0.01	
Communication				
Baseline	33 ± 11	33 ± 9.6	33 ± 11	36 ± 11
Endpoint	27 ± 12	26 ± 11	29 ± 11	33 ± 10
Change	−6.4 ± 8.8	−6.5 ± 7.8	−4.3 ± 10	−3.0 ± 7.0
*P*-value ^1^	0.07	0.10	>0.1	
Effect size ^2^	0.07	0.06	0.05	
Social motivation				
Baseline	16 ± 6.8	16 ± 4.9	13 ± 5.6	15 ± 6.0
Endpoint	13 ± 6.9	12 ± 5.9	11 ± 5.5	14 ± 4.0
Change	−2.8 ± 3.9	−4.3 ± 4.2	−1.5 ± 4.7	−1.3 ± 4.2
*P*-value ^1^	>0.1	0.05	>0.1	
Effect size ^2^	0.03	0.09	<0.01	
Repetitive and restricted interest and behaviour				
Baseline	19 ± 6.5	20 ± 6.9	18 ± 6.1	22 ± 6.0
Endpoint	16 ± 5.5	16 ± 8.2	17 ± 7.7	20 ± 6.0
Change	−3.8 ± 4.6	−3.8 ± 4.8	−1.4 ± 5.5	−1.8 ± 5.6
*P*-value ^1^	>0.1	>0.1	>0.1	
Effect size ^2^	0.04	0.03	<0.01	

VID, vitamin D; OM, omega-3; VIDOM, vitamin D + omega-3; SRS, Social Responsiveness Scale. Values are reported as mean ± SD. ^1^ Pair-wise mixed effects models. The analyses were adjusted for therapy, medication, and compliance over the study period. ^2^ Partial eta-squared η_p_^2^; 0.01 = small effect size; 0.06 = medium effect size; 0.14 = large effect size.
